# Micro-concentration Lipopolysaccharide as a Novel Stimulator of Megakaryocytopoiesis that Synergizes with IL-6 for Platelet Production

**DOI:** 10.1038/srep13748

**Published:** 2015-09-02

**Authors:** Di Wu, Jun Xie, Xuejun Wang, Bingcheng Zou, Yin Yu, Tao Jing, Songmei Zhang, Qing Zhang

**Affiliations:** 1 State Key Laboratory of Biocontrol, School of Life Sciences, Sun Yat-sen University, Guangzhou, China; 2 Key Laboratory of Tropical & Subtropical Fishery Resource Application & Cultivation, Ministry of Agriculture, Pearl River Fisheries Research Institute of CAFS, Guangzhou, China; 3 Institute of Sun Yat-Sen University in Shenzhen, Shenzhen, China

## Abstract

Lipopolysaccharide (LPS) induces platelet activation and enhances platelet sensitivity to aggregation, which might alter platelet counts. We found that serial doses of micro-concentration LPS significantly increased the platelet count in mice treated with kanamycin, along with increased expression of IL-6 compared with IL-3 and TPO in megakaryocytes obtained from the mouse bone morrow following LPS administration. Furthermore, LPS at lower levels ranging plus IL-6 effectively stimulated CFU-MK formation and increased CD41 expression and megakaryocyte polyploidization. Meanwhile, there was a sustained rise in the percentage of reticulated platelets in the whole blood in response to low-dosage LPS combined with IL-6. *In vivo* experiments also demonstrated that the administration of LPS combined with IL-6 substantially enhanced the number of circulating platelets in normal and thrombocytopenic mice. Notably, the optimal LPS concentration in combination with IL-6 might be a novel stimulator of TLR4 and IL-6R expression in Dami cell lines, which initially occurs through TLR4-IL-6R crosstalk and then involves the activation of NF-κB and phosphorylation of p38 MAPK. These data suggest a new paradigm for the regulation of megakaryocytopoiesis and platelet production via a synergistic effect of LPS and IL-6, which has the potential to be used for the design of new therapies.

Mammals, including humans, are often in contact with Gram-negative bacteria and bacterial lipopolysaccharide (LPS). Consequently, deadly bacterial strains may have developed evolutionary advantages, such as the physiological regulation of their hosts. Furthermore, they possess potent machinery to stimulate the production of a variety of immune and hematopoietic mediators. Low levels of LPS are thought to be beneficial for the host because they can cause immune stimulation and enhanced resistance to infections and malignancies^1^. However, the release of large quantities of LPS into the bloodstream is clearly deleterious to the host, and this event can precipitate the induction of a potentially lethal array of inflammatory mediators and procoagulant factors^2^,^3^. Meanwhile, molecules involved in the immune response are also activated; LPS-induced cytokines, including interleukin 1 (IL-1), IL-3, and IL-6, regulate the host responses to infection, immune responses, and inflammation^4^,^5^. Among these molecules, IL-6 shows a prominent megakaryocytopoiesis-promoting effect and mediates pro-inflammatory responses^6^,^7^. Notably, the crosstalk between LPS and IL-6 has thus far proven to be mediated by rapid nuclear factor-kappaB (NF-κB) activation, dissociation from the inhibitor and translocation to the specific DNA-binding site^8^. Although IL-6 contributes significantly to megakaryocytopoiesis, IL-6 cannot restore platelet production in Tpo^–/–^ or Mpl^–/–^ mice^9^, and IL-6 stimulation alone may be insufficient to initiate platelet production. Therefore, thrombopoietin (TPO) is required to co-regulate megakaryocyte maturation and thrombopoiesis^10^, which can promote megakaryocyte endomitosis and maturation when the spindles fail to separate, resulting in polyploidy^11^,^12^. The increased polyploidization of megakaryocytes is necessary to induce the further development of the cells into reticulated platelets (RPs) until a massive endoplasmic reticulum is formed^10^, which can then be used to increase the production rate of new platelets^13^. In addition to polyploidization, CD41/CD61 expression functions as a marker of megakaryocyte and platelet maturation^14^. IL-6 can also remarkably enhance the growth-promoting effect of TPO on megakaryocytes^15^. However, few studies have been conducted to determine whether megakaryocyte stimulation by LPS alone and/or in combination with IL-6 occurs via a novel synergistic effect.

Gram-negative bacteria release LPS, which activates Toll-like-receptor-4 (TLR4) in the host, initiating an inflammatory response to infection. TLR4 is a trans-membrane glycoprotein expressed by leukocytes, lymphocytes, megakaryocytes, and platelets that allows platelets to recognize bacterial proteins and regulate platelet immunity and function^16^,^17^. Further studies have shown that LPS-induced TLR4 stimulation in bone marrow cells activates a signaling cascade that is characterized by pro-inflammatory cytokine production and a subsequent immune response^18^,^19^. Moreover, the effect of inflammation on the regulation of platelet production and function is related to TLRs in platelets and megakaryocytes^20^. In addition, the *in vivo* effects of LPS and TLR4 on platelet production and activity can increase thrombotic risk^17^. Considering that platelets do not contain any nuclei to regulate cell division, we hypothesized that such increased platelet production may be attributed to megakaryocytes. However, few studies have examined the expression and function of TLRs in megakaryocytes and whether these receptors could have a role in platelet production^16^. Recent results have shown that TLR4 expression increases as megakaryocytes mature, suggesting that platelets could acquire expression of this receptor during their genesis as protoplatelets from megakaryocyte membranes^21^. We considered that megakaryocytes may be further influenced by pro-inflammatory cytokines from either autocrine or paracrine signaling and later differentiate into RPs as the megakaryocytes become more susceptible to LPS due to increased TLR4 expression. In contrast to megakaryocytes, platelets function as potential immune modulators in host defense responses and sepsis, in which platelets exhibit a pro-thrombotic phenotype in response to very low LPS concentrations^16^. Moreover, exposure to low doses of LPS could increase the levels of TPO and cytokines, which are important for thrombopoiesis and contribute to a corresponding increase in RPs^16^,^22^. These results may also provide insight into the relationship between LPS- or infection-associated platelet production and TLR4-dependent pathways. Thus, the *in vivo* mechanisms of the priming effect of low LPS doses on megakaryocyte development and platelet production should be further determined.

To investigate the possibility that platelet production could be stimulated by the possible synergistic effect of IL-6 and micro-concentration LPS, we examined the megakaryocyte colony forming units (CFU-MK), megakaryocyte CD41 expression, polyploidization and percentages of RPs following LPS and IL-6 treatment. Furthermore, we investigated whether this megakaryocyte-related cell development may result in platelet alterations in normal and thrombocytopenic mice stimulated with IL-6 plus LPS. Our results suggest that TLRs may be the key link in the megakaryocytopoiesis communication network, incorporating IL-6R upregulation following LPS and IL-6 administration. To the best of our knowledge, the present study is first to provide evidence that trace amounts of LPS, functioning as a novel stimulator, can synergize with IL-6 to act on megakaryocytopoiesis and platelet production.

## Materials

### Cytokines and reagents

Murine IL-6 and TPO were obtained from R&D Systems (Minneapolis, MN, USA). LPS (from *Escherichia coli* 055:B5); PD98059, a specific inhibitor of ERK kinase (MEK1/2); SB203580, a specific inhibitor of p38 kinase; pyrrolidinecarbodithioic acid (PDTC), an inhibitor of NF-kB, and 5-fluorouracil (5-FU) were purchased from Sigma-Aldrich (St. Louis, MO, USA). Anti-phospho-ERK1/2 mAb, anti-phospho-p38 MAPK mAb, TLR4 monoclonal antibodies, IL-6R monoclonal antibodies and peroxidase-conjugated secondary antibody were purchased from Santa Cruz Biotechnology, Inc. (Santa Cruz CA, USA).

### Animals and cell culture

Adult male BALB/c mice were purchased from the breeding facilities of the School for Medical Research, Sun Yat-sen University (Guangzhou, China). The femurs of the mice were removed for processing and subsequent cell preparation. Bone marrow cells were removed from the femurs using Isocove’s modified Dulbecco′s medium (IMDM, Gibco, USA). Pooled cell suspensions were prepared in IMDM supplemented with 1% Nutridoma (Roche, Indianapolis, IN, USA). All experimental protocols were approved by the Committee on the Use of Live Animals in Sun Yat-sen University. The methods were conducted in accordance with the approved guidelines.

### Experimental design

BALB/c male mice under conventional conditions were treated with kanamycin (5.0, 50.0 mg/day; Shenggong Ltd., China) for 10 days via intragastric administration. Meanwhile, mice were injected intraperitoneally (IP) with 0.1, 1.0, 10.0, 100.0 and 1000.0 μg of LPS or 100.0 μg of IL-6 per 100 g of body weight. Platelets were counted using a F-820 Sysmex electronic blood cell analyzer (Sysmex Corp Ltd., Japan).

### Enzyme-linked immunosorbent assay (ELISA) and semi-quantitative RT-PCR

IL-3, IL-6, and TPO serum levels in the culture media were quantified using ELISA kits (R&D Systems, Minneapolis, MN, USA). Immature and mature megakaryocytes from the samples supplemented with LPS were separated using a discontinuous BSA density gradient (0%, 1.5%, and 3.0% in PBS). Total cellular RNA was isolated using TRIzol according to the manufacturer’s instructions. cDNA was used for PCR amplification with the following primer pairs: 5′-TACCACTGGCATCGTGATGGACT-3′ and 5′-TCCTTCTGCATCCTGTCGGCAAT-30′ as β-actin sense and antisense primers,respectively; 5′-GCTCCCATGACCCAGACAACGTCC-3′ and 5′-CAGATAGAACGTCAGTTTCCTCCG-3′ as IL-3 sense and antisense primers, respectively; 5′-AAATGCCAGCCTGCTGACGAAC-3′ and 5′-AACAACAATCTGAGGTGCCCATGCTAC-3′ as IL-6 sense and antisense primers, respectively; 5′-TGAGCAGGCTTCAGGGATTC-3′ and 5′-CCCAGGGCCTTTTAGGTGAA-3′ as IL-6 sense and antisense primers, respectively; and 5′-CGTTTCCTGATGCTTGTAGG-3′ and 5′-GAAGGAGAAATCAGGCTG-3′ as TPO sense and antisense primers, respectively. The amount of cDNA was normalized to the intensity of the PCR products of β-actin. All the PCR products were quantified with Quantity One software (version 4.6.2, Bio-Rad Laboratories) and expressed as relative absorbance units. The RT-PCR was performed in triplicate.

### *In vitro* determination of CFU-MKs

Suspensions containing 5 × 10^5^ bone marrow cells were plated in IMDM supplemented with 1% Nutridoma and solidified with 0.4% agar in the presence of LPS alone (0.1, 10.0, 1000.0 ng/ml), IL-6 alone (50.0 ng/ml) or both together to determine the number of CFU-MKs for 7 d at 37 °C in a fully humidified atmosphere containing 5% CO_2_. The plates were stained to determine the acetylcholinesterase activity. The experiments were performed in triplicate.

### Flow cytometry

Mice were injected IP with TPO (1.0 μg/kg/day) for 5 d. The low-density bone marrow cells from TPO-pretreated mice were isolated as described previously and cultured in the presence of IL-6 (30.0 ng/ml) either alone or combined with LPS (0.1, 10.0, and 1000.0 ng/ml). The cells were incubated with FITC-conjugated CD41 antibody for 20 min at room temperature to identify the megakaryocytes. The DNA content of these megakaryocytes was then determined by staining with propidium iodide (PI). Cells expressing CD41 and their DNA content were quantified using a FACS Vantage flow cytometer (Becton Dickinson) and Cell Quest software.

### Assessment of ERK1/2 and p38 MAPK

The lysed Dami cells were separated by SDS-12% PAGE in buffer (25 mM Tris, 250 mM glycine, and 0.1% SDS), and the proteins were electroblotted onto Protran nitrocellulose transfer membranes (Schleicher & Schuell Inc., Keene, NH, USA). The blots were probed with anti-phospho-ERK1/2 mAb or anti-phospho-p38 MAPK mAb for 1 h, as described by the manufacturer of the antibodies. Next, the membranes were incubated with secondary HRP-conjugated anti-mouse IgG for 1 h. The blots were visualized using SuperSignal West Femto Maximum Sensitivity Substrate as described by the manufacturer (Pierce, Rockford, IL).

### Flow cytometric measurement of RPs

Adult blood was collected in a heparinized 1 ml syringe following cardiac puncture of the sacrificed mice treated with 100.0 μg/kg IL-6 alone, 0.8 μg/kg LPS alone or IL-6 combined with 0.8 μg/kg or 80.0 μg/kg LPS. Approximately 5.0 μl of whole blood was added to a tube containing 1.0 ml of 10.0% thiazole orange (Sigma). Flow cytometry was performed for 1 h to count 5,000 stained platelets within the platelet gate. The platelets demonstrating an increase in the mean fluorescence intensity beyond a threshold margin of 1.0% of the total platelets at baseline were counted as RPs. RPs were expressed as the percentage of the total counted platelets.

### Quantitative-PCR (Q-PCR) and western blot analysis of TLR4 and IL-6R

TLR4 and IL-6R were differentially quantified according to a standard protocol using SYBR green and the ABI 7700 sequence detection system (Applied Biosystems, CA). The sequences of the specific primers are as follows: 5′-GCAATTTCCTGCGCACTACA-3′ (sense), 5′-GGAAGCGAGGGCCACAA-3′ (antisense), and 5′-GAGACTGAGGCATGC-3′ for the exon 10/11 boundary of TLR4; 5′-AGCGAGGGCCACAAAGC-3′ (sense), 5′-CAGCTCAAGAGACCTGCTACCA-3′ (antisense), and 5′-CCTCAGTCTCCTTCCAGT-3′ for IL-6R.

∆*C*_T_ values were derived by subtracting the threshold cycle (Ct) values of TLR4 and IL-6R from the ∆*C*_T_ value of the ribosomal protein L19, which was used as an internal control. All the reactions were performed in duplicate. For transient transfections in human kidney 293T cells, TLR4 and IL-6R cDNA was subcloned into the pcDNA3 expression vector (Invitrogen) as XhoI-NotI fragments. The 293T cells were transiently transfected with varying amounts of plasmid DNA encoding TLR4 and IL-6R and then analyzed at 48 h post-transfection. The total amounts of TLR4 and IL-6R were determined by immune blot analysis using monoclonal antibodies directed against TLR4 and IL-6R. To control for protein loading, we re-probed the membranes using the mouse monoclonal antibody AC-40, directed against actin (Sigma).

### NF-κB luciferase and transcription factor assay

Dami cells (5 × 10^7^) were co-transfected with an NF-κB-Luc reporter plasmid plus the phRL-TK plasmid (Promega, WI, USA) using Lipofectamine LTX™ (Invitrogen, CA, USA) according to the manufacturer’s instructions. After 12 h of transfection, the cells were pretreated with IL-6 either alone or combined with LPS (0.1, 10.0, and 1000.0 ng/ml) for 48 h. Then, the luciferase activity of lysed cells was determined using the Promega luciferase assay system (Promega, CA, USA). Nuclear proteins were extracted using the active motif nuclear extract protocol (Active Motif, Carlsbad, CA, USA). The NF-κB p65 subunit was activated in 3 μg of nuclear extract, and this activation was verified using an NF-κB p65 ELISA-based transcription factor assay kit (TransAM™ assay; Active Motif, CA, USA) according to the manufacturer’s instructions. The NF-κB-detecting antibody recognizes an epitope on p65 that is accessible only when NF-κB is activated. The colorimetric reading at 450 nm was determined using a microplate reader (Versa Max; Molecular Devices, CA, USA).

### *In vivo* experiments

BALB/c mice were injected IP with IL-6 (100.0 μg/kg/day) for 7 d either alone or in combination with varying doses of LPS (0.8, 8.0 or 80.0 μg/kg/day). PBS containing 100 μg/ml BSA was used as the negative control treatment. Venous blood (20.0 μl) was collected from a small lateral cut in a tail vein at 0, 4, 7, 10, 13, and 16 d. For platelet counts in the mice with thrombocytopenia, adult mice were injected IP with 5-FU at a dose of 150.0 mg/kg body weight. One hour later, these animals were divided into four groups and received IP PBS-BSA buffer, 100.0 μg/kg/day IL-6, 0.8 μg/kg/day LPS or 100.0 μg/kg/day IL-6 plus 0.8 μg/kg/day LPS for 7 d. Platelets were counted on days 0, 4, 7, 10, 13, 16, and 19. Control animals were injected with PBS-BSA instead of receiving the cytokine treatments. Another set of mice did not receive the 5-FU treatment but still received the PBS-BSA injections.

### Statistical Analysis

The data are presented as the mean ± standard deviation from at least 3 separate experiments. Unless otherwise noted, the differences among groups were analyzed using Student’s *t* test when only two groups were compared or by one-way analysis of variance (ANOVA) when more than two groups were compared. All tests were two-sided. Differences were considered to be statistically significant at *P < 0.05, **P < 0.01, and ***P < 0.001.

## Results

### Effects of LPS on peripheral blood cells

**Figure 1:**
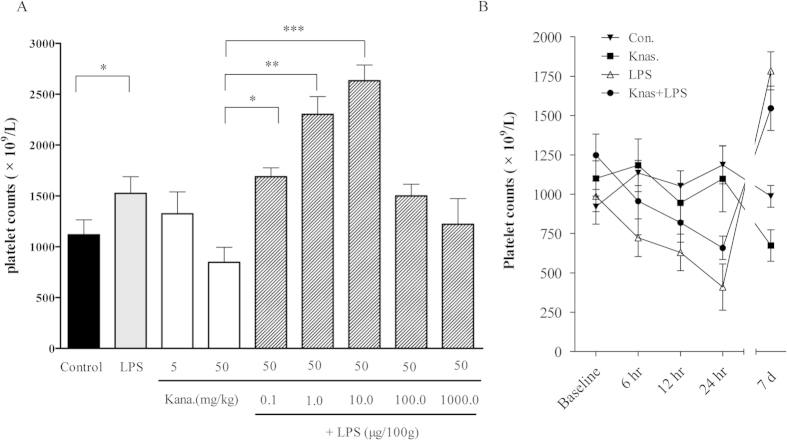
Effect of LPS on circulating platelet counts in mice. (**A**) Circulating platelets in kanamycin-treated (5, and 50 mg/day) and kanamycin plus LPS-treated (0.1, 1.0, 10.0, 100.0 and 1000.0 μg/100 g/day) mice for 10 d. Platelet counts with LPS IP (0.1, 1.0 and 10.0 μg/100 g/day) were significantly increased compared with the control (*P* < 0.05) in a dose-dependent manner. (**B**) The respective time curve represents the mice recorded at 3, 6, and 24 h and 7 d after the termination of treatments. Platelet counts slightly decreased after 7 d of kanamycin injection. The IP injection of LPS restored the platelet numbers after 7 d in kanamycin-pretreated mice. In contrast, LPS reduced the circulating platelet count in 24 h.

To eliminate LPS release from Gram-negative microbes in the gastrointestinal tract and then simulate an endogenous LPS effect, Balb/c mice were irrigated with kanamycin and injected IP with different concentrations of LPS for 10 d. The dose-dependent effects of LPS on the peak and time-dependent parameters of platelet production were examined to find the appropriate dose. A trace amount of 0.1, 1.0, and 10.0 μg/100 g/day LPS could lead to an increase in the platelet count in a dose-dependent manner, with a plateau at 211% higher than in the kanamycin-treated groups with an average platelet count of 2633 × 10^9^ /L. Additionally, we observed a slight decrease in the number of circulating platelets after one week when the mice were injected with 50 mg/ml kanamycin. However, the same results showed that the treatment with 10.0 μg/100 g/day exogenous LPS alone without the intragastric perfusion of kanamycin could increase the platelet counts compared with the control groups (Fig. 1A). This result is consistent with physiological conditions, in which low LPS concentrations circulate in the human bloodstream without initiating severe inflammation^23^. Meanwhile, the number of circulating platelets in mice injected with trace amount of LPS alone or LPS plus kanamycin were higher than the number of platelets in control mice, with both groups exceeding the pre-treatment levels at 7 d (*P* < 0.01). However, the number of circulating platelets decreased at 24 h post-LPS injection compared with the control group (*P* < 0.05) (Fig. 1B), suggesting that platelet production occurs in response to the long-term effects of LPS plus IL-6. Furthermore, it was shown the dynamic changes of platelet counts of mice after the IP injection of LPS, Kana, Kana plus LPS and NS as control. The platelet counts of control group mice in different times were not significantly different (ANOVA, *P* > 0.05), while those of mice in three treatments showed significant time-related changes (ANOVA, *P* < 0.05, 0.05 and 0.01 for Kana, LPS and Kana plus LPS respectively). In addition, significant difference of platelet counts between different treatments was revealed by MNOVA (*P* < 0.01), showing as the platelet counts of mice treated by LPS and Kana plus LPS significantly higher than those of Kanamycin treatment and control group. These studies indicated that trace LPS from enteric bacteria could play a key role in the regulation of platelet production in mice.

### Effect of LPS on IL-3, IL-6 and TPO expression in kanamycin-treated mice

**Figure 2:**
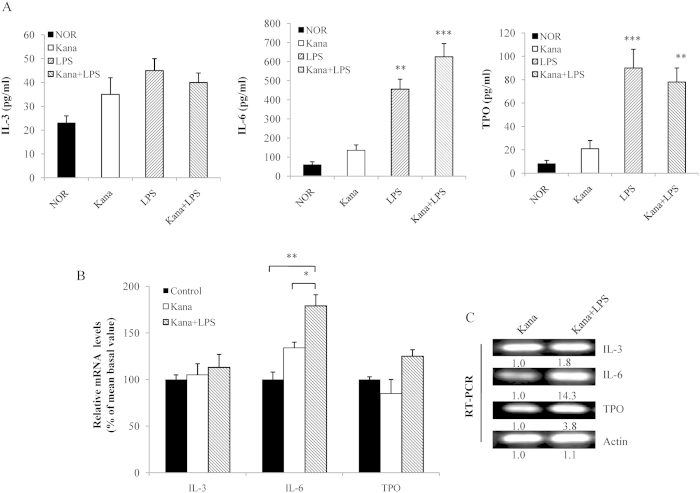
Levels of serum and mRNA expression of IL-3, IL-6 and TPO. (**A**) The serum IL-3, IL-6, and TPO levels were measured by ELISA after LPS injection for 7 d in kanamycin-treated mice. Cells belonging to different stages of megakaryocyte cell maturity from the isolated bone marrow were separated on discontinuous BSA gradients. Significant results compared with the control are based on *P < 0.05 or **P < 0.01. (**B**) Relative mRNA levels of the megakaryocyte subpopulations were assessed by quantitative RT-PCR following treatment with kanamycin or kanamycin plus LPS. Two sets of 10 mice were used to determine the basal platelet levels. (**C**) RT-PCR was performed using total RNA obtained from immature and mature megakaryocytes with primers specific for IL-3, IL-6, and TPO. The fragments were visualized by ethidium bromide staining after separation. The numbers below the blots represent the relative expression levels.

Considering that LPS can recover platelet production in kanamycin-treated mice, we investigated the effects of LPS on the expression levels of IL-3, IL-6 and TPO. LPS (100.0 μg/kg, IP injection) markedly increased the serum IL-6 and TPO levels, whereas the serum IL-3 level was not significantly affected (Fig. 2A). Next, we evaluated the *in vivo* effects of LPS by monitoring the IL-3, IL-6, and TPO expression levels in megakaryocytes by semi-quantitative RT-PCR. The mice received kanamycin as a control treatment. Another set of mice received LPS together with kanamycin. The megakaryocytes were then purified into cell fractions based on size using discontinuous BSA gradients. The results showed that the mRNA levels of IL-6 were significantly higher than those of IL-3 and TPO in the different groups (Fig. 2B). Although the mRNA levels of IL-3 and TPO in the group treated with kanamycin together with LPS were higher than those in the kanamycin group, only the increase in the IL-6 mRNA was significant (Fig. 2B,C). Thus, the up-regulated expression of IL-6 seems to be directly correlated with the initial response to LPS stimulation.

### Effects of LPS in combination with IL-6 on megakaryocyte development

**Figure 3:**
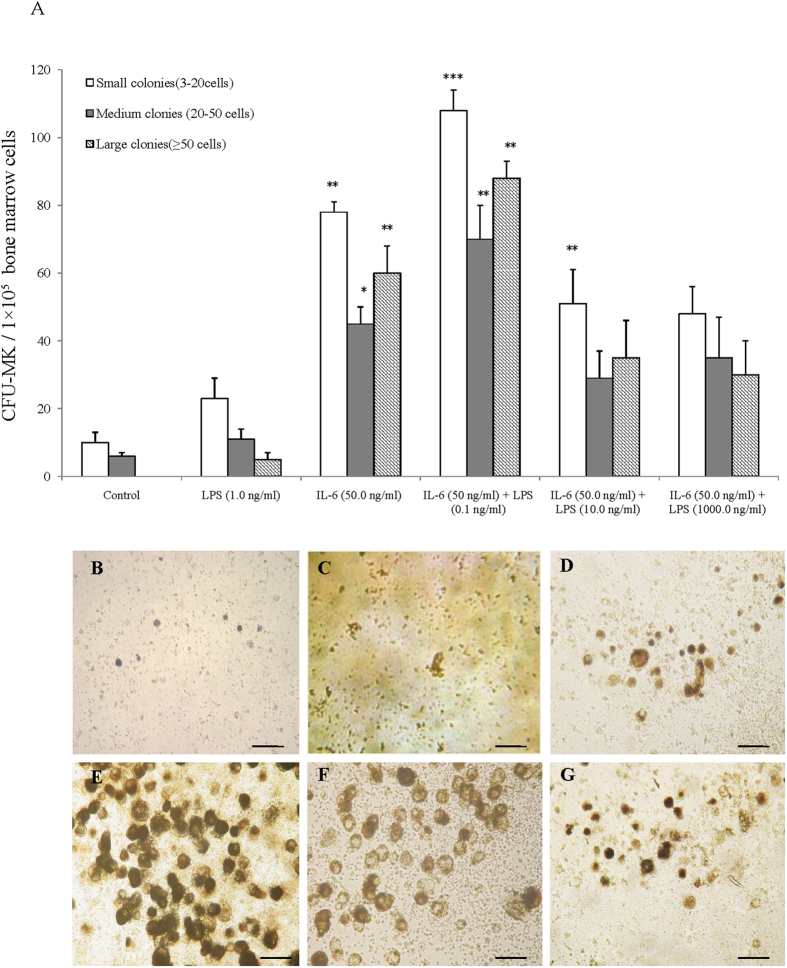
Effect of LPS alone or with IL-6 on CFU-MKs. (**A**) Number of CFU-MKs in different mouse groups after incubation with LPS alone or IL-6 with LPS. Colonies classified according to their size were evaluated by inverted light microscopy. Experiments were performed in triplicate in four assays. Significant results compared with PBS are based on *P < 0.05 or **P < 0.01. (**B**) Bone marrow cells from TPO-pretreated mice were isolated by a discontinuous BSA density gradient and grown in a liquid serum-free medium in LPS alone or with IL-6 for 3 d. Cells were stained for acetylcholinesterase activity and phase contrast morphology of megakaryocyte cells. Scale bar, 10 μm.

The *in vitro* levels of CFU-MK in a serum-free, semi-solid culture of murine bone marrow with different LPS concentrations (0.1 ng/ml to 1000.0 ng/ml) and IL-6 (50.0 ng/ml) were tested to determine the appropriate doses. The plateau levels of the dose-response curves were obtained at 50 ng/ml for IL-6 and 0.1 ng/ml to 10 ng/ml for LPS (data not shown). The proportions of CFU-MKs, estimated from the colonies containing 2 to 20 cells, 21 to 50 cells, and >50 cells, increased when the cells were exposed to IL-6 alone (20 ng/ml) or IL-6 with LPS (0.1 and 10 ng/ml). The treatment containing 50.0 ng/ml of IL-6 and 1000.0 ng/ml of LPS markedly reduced the number of CFU-MKs compared with the treatment with IL-6 only or IL-6 plus 0.1 or 10.0 ng/ml LPS (Fig. 3A). The majority of the increased number of CFU-MKs in IL-6 plus LPS treated cells were 3-20 cells/colony, whereas the number of CFU-MKs (>50 cells/colony) reached their highest level in the presence of IL-6 plus LPS (0.1 ng/ml). We next examined the morphological differences between the different stimulants. The megakaryocytes stimulated with 50 ng/ml IL-6 plus 0.1 ng/ml LPS had a more heavily stained cytoplasm than those stimulated with IL-6 alone, LPS alone or IL-6 (50.0 ng/ml) plus 10.0 or 1000.0 ng/ml LPS (Fig. 3C,E,F). The greater cell size with a heavily stained cytoplasm was also observed in the cells treated with 50.0 ng/ml IL-6 plus 0.1 ng/ml LPS (Fig. 3D). There were no other larger colonies, and a significant difference was observed among the other groups in these media based on microscopy analysis (Fig. 3B,E,F).

### Effects of LPS in combination with IL-6 on megakaryocyte maturation

**Figure 4:**
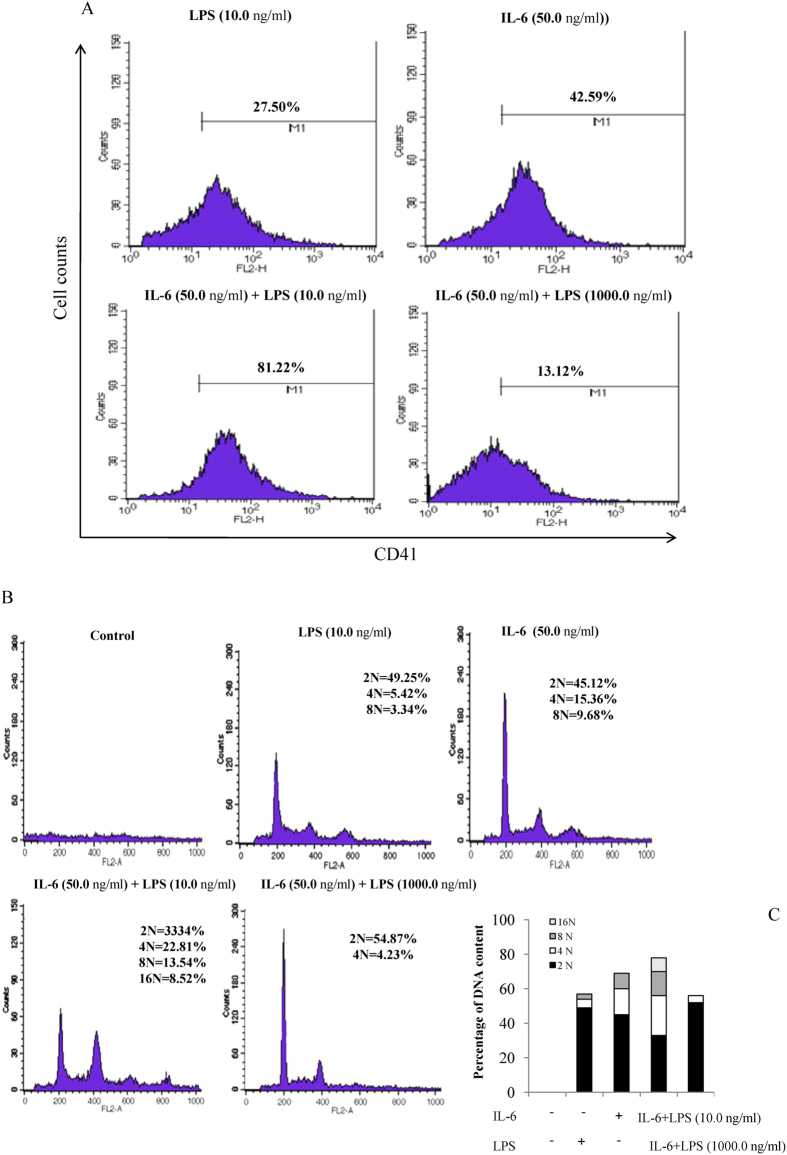
Effects of LPS alone or with IL-6 on CD41 expression and polyploidization. (**A**) Bone marrow cells were grown in a liquid serum-free medium supplemented with LPS or IL-6 alone or together for 3 d. Megakaryocytes were stained with FITC-conjugated anti-mouse CD41 monoclonal antibody and analyzed by FACS with large forward-scatter and side-scatter properties. The experiment was repeated three times, and similar results were obtained. (**B**) Bone marrow cells from TPO-pretreated mice were isolated on a discontinuous BSA density gradient and grown in a liquid serum-free medium in the presence of IL-6 or LPS alone or together for 3 d. The flow cytometric analysis of the DNA content of CD41-positive cells was examined by PI staining. The experiment was repeated three times, and similar results were obtained. (**C**) The histogram plots of PI staining were calculated from the percentage of DNA content.

To determine the effect of LPS and IL-6 on the terminal differentiation of megakaryocytes, the cells were also analyzed to investigate CD41 expression by flow cytometry. Low-density bone marrow cells from normal mouse cells were incubated either with 50.0 ng/ml IL-6 or with IL-6 combined with different doses of LPS for 3 d. The results of direct FITC fluorescence staining and flow cytometric analysis of CD41 indicated the percentage of cells undergoing megakaryopoiesis. Figure 4A shows that only 27.50 ± 3.3% and 42.59 ± 7.1% of the cells were CD41 positive in the presence of LPS (10.0 ng/ml) and IL-6 (50.0 ng/ml), respectively. However, the CD41-positive cells showed significant changes in the cultures treated with LPS and IL-6. The number of CD41-positive cells in the IL-6 (50.0 ng/ml) plus LPS-supplemented (10.0 ng/ml) culture was higher (81.22 ± 8.4%) than that observed in the IL-6 alone or IL-6 plus 1000.0 ng/ml LPS cultures (13.12 ± 5.6%, ). We also examined the nuclear DNA content of the CD41-positive cells stained with PI to determine the polyploidization of the treated cells. Figure 4B shows that a significant populations of the CD41-positive cells were 4N (49.25 ± 3.3%) and 4N (45.12 ± 2.5%) after the bone marrow cells were treated with alone LPS or IL-6. However, the megakaryocyte ploidy number was either 4N (22.81 ± 4.5%) or 8N (13.54 ± 2.1%) in the IL-6 plus 10.0 ng/ml LPS culture, with 16N (8.52 ± 3.7%) megakaryocytes being also observed. Conversely, a significantly decreased population of megakaryocytes was produced in IL-6-treated cultures with 1000.0 ng/ml LPS; these megakaryocytes contained a DNA content of 2N and 4N (54.87 ± 2.6% and 4.23 ± 1.8%, respectively). It should be noted that a significant population of makaryocytes produced in IL-6 plus LPS (10.0 ng/ml)-contained cultures had relative higher counts in term of 4N DNA content compared with the other treatments, which was coupled with an increase in the population of cells in 8N DNA content (Fig. 4C).

### Detection of RPs in the whole blood of mice

**Figure 5:**
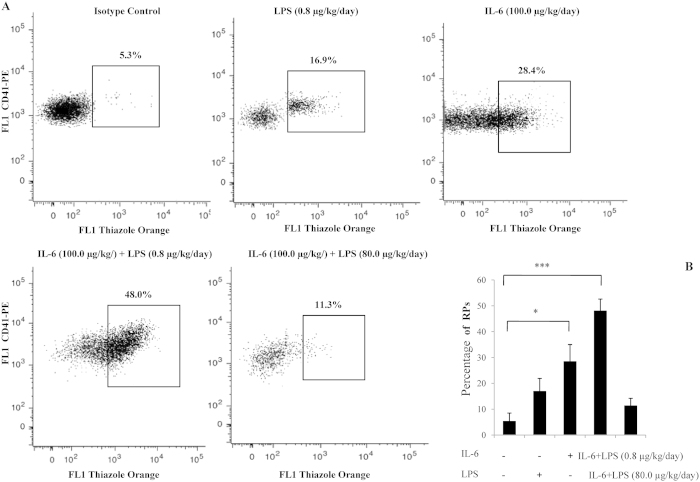
Detection of RPs in the whole blood of mice. (**A**) Platelet fractions were identified by FACS forward-scatter and side-scatter parameters. The mean fluorescence intensity >1% of all platelets at baseline was used to set the threshold of the thiazole orange signal by analyzing 5000 platelets. Dot plots show the distribution of thiazole-positive events within the platelets in the whole blood. Regions are set on the RP percentage for blood samples from mice receiving LPS or IL-6 treatment alone or from LPS- plus IL-6-treated mice. The flow cytometric data were analyzed using a flow cytometer (Becton Dickinson) and CellQuest software. (**B**) Histogram showing the percentage of RPs stimulated by LPS and IL-6 in three independent experiments. *P < .05; **P < 0.01.

The RP population generally represents younger platelets with an increased mean volume and a greater number of dense granules than older circulating platelets^24^. Under control conditions (prior to LPS treatment), the percentage of RPs (containing mRNA) was significantly less than that in mice treated with IL-6 or IL-6 plus LPS. Approximately 16% and 7% of maternal platelets were reticulated following the IP injection of 100.0 μg/kg/day IL-6 and 0.8 μg/kg/day LPS for 7 d, respectively. However, more than 43% of the platelets were reticulated in the presence of IL-6 plus LPS. Unexpectedly, thiazole orange staining indicated that the number of RPs following 100.0 μg/kg/day IL-6 plus 80.0 μg/kg/day LPS treatment decreased to 5% of the platelets (Fig. 5A). Although the RPs levels of IL-6 alone or IL-6 plus LPS (0.8 μg/kg/day) was significantly more than that for the control, the RPs levels of IL-6 plus LPS (80.0 μg/kg/day) approached those seen in the control (Fig. 5B) Thus, the increased production of platelets could be partially related to the continually expanding megakaryocytic polyploidy at suitable stimulating doses of LPS plus IL-6. These results suggest that the continually expanding rnRNA synthesis during megakaryocyte maturation could lead to the production of platelets following LPS plus IL-6 administration.

### TLR4 mediates a dose-dependent increase in IL-6R

**Figure 6:**
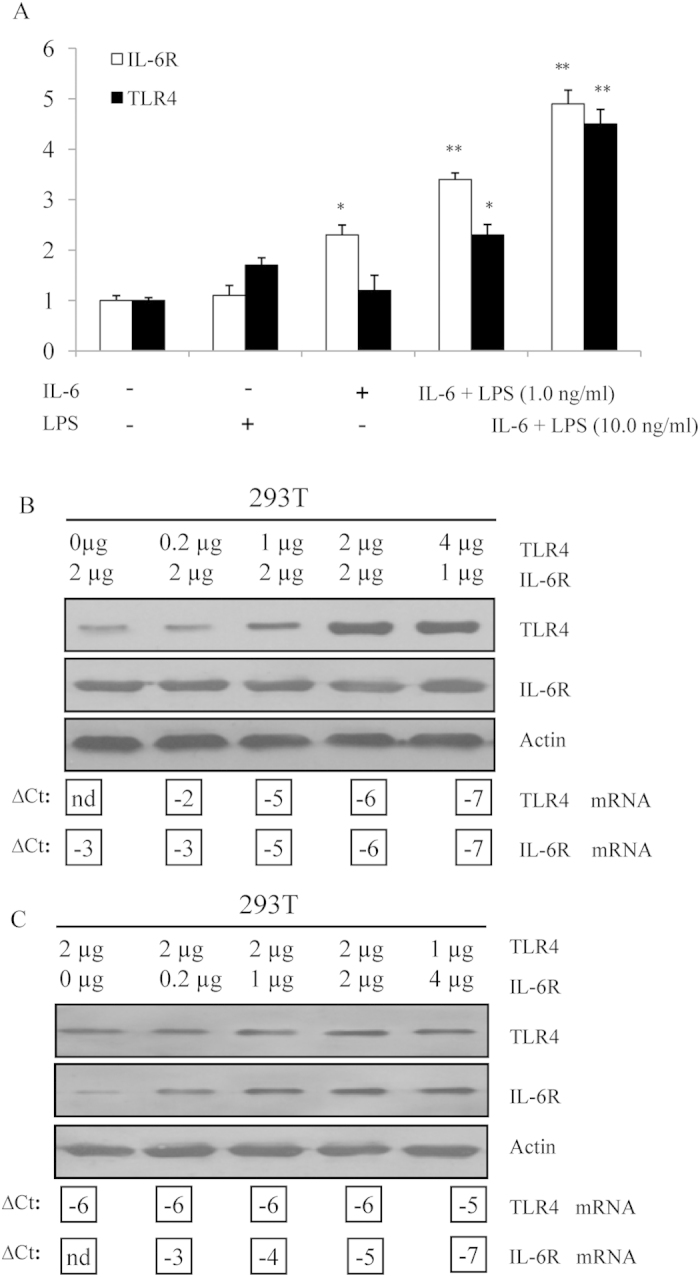
Dose-response relationships for TLR4 expression with IL-6R expression. (**A**) Alteration in the expression of the TLR4 and IL-6R genes. Total RNA was extracted from Dami cells treated with LPS alone, IL-6 alone or LPS plus IL-6 for 48 h. The relative expression levels of TLR4 and IL-6R in the treated Dami cells were compared with those in the untreated Dami cells. All measurements show the expression relative to the expression levels of β-actin. The values are based on three independent experiments with triplicate measurements for each. **P < 0.01; ***P < 0.001. (**B**) 293T cells were transiently co-transfected with varying amounts of plasmid DNA encoding TLR4 and IL-6R. TLR4, IL-6R, and actin proteins were detected by immunoblot analysis. The mRNA expression of TLR4 and IL-6R was determined by Q-PCR; ∆CT values for TLR4 and IL-6R are shown below the corresponding lanes of the immunoblots. The amount of TLR4 DNA was kept constant, whereas the amount of IL-6R DNA was varied. (**C**) TLR4 DNA was varied, and the amount of IL-6R DNA was kept constant.

Based on the TLR expression and IL-6 secretion in megakaryocytes, LPS might be involved in the TLR-mediated IL-6/IL-6R signaling pathway. First, we asked whether TLR4 and IL-6R protein surface expression was affected by the presence of LPS plus IL-6. Dami cells were exposed to small amounts (1.0 and 10.0 ng/ml) of LPS in the presence of 50.0 ng/ml IL-6 to measure the relative activation of TLR4 and IL-6R in treated compared with untreated cells. Although IL-6 by itself increased IL-6R activation 2.3-fold in Dami cells, co-incubation with 1.0 ng/ml and 10.0 ng/ml LPS increased TLR4 and IL-6R activation 4.5 and 4.9-fold, respectively (Fig. 6A). The increase in TLR4 activation could be attributed to IL-6R up-regulation followed by an LPS synergistic effect compared with the responses to IL-6 alone. Furthermore, we transiently co-transfected 293T cells with a constant amount of IL-6R-encoding plasmid and varying amounts of plasmid encoding TLR4 to investigate LPS-induced megakaryocytopoiesis by regulating the cross-talk of TLR4/IL-6R signaling. In this experiment, the amount of IL-6R protein was affected by TLR4 in a dose-dependent manner. Measuring the mRNA levels by Q-PCR confirmed that mRNA expression also correlated with the amount of transfected plasmid DNA. The TLR4 (∆*C*_T_) value for cells transfected with 0.2 μg of TLR4 was –3. Cells transfected with 20 times the amount of TLR4 (4 μg) had a TLR4 (∆*C*_T_) value of –7. This difference corresponded to a decrease in 4 ∆*C*_T_, which is equal to 2^4^ = 16 times higher TLR4 expression (Fig. 6B). In the converse experiment, 293T cells were co-transfected with a constant amount of TLR4 and different IL-6R concentrations. The stable TLR4 expression was not altered by the presence of increasing amounts of IL-6R (Fig. 6C). This result suggests that the unidirectional cross-activation of IL-6R by TLR4 reflects a stable association between the LPS inducer and the enhanced IL-6 response.

### LPS synergizes with IL-6 to phosphorylate p38 MAPK and regulate TLR4 and IL-6R mRNA expression

**Figure 7:**
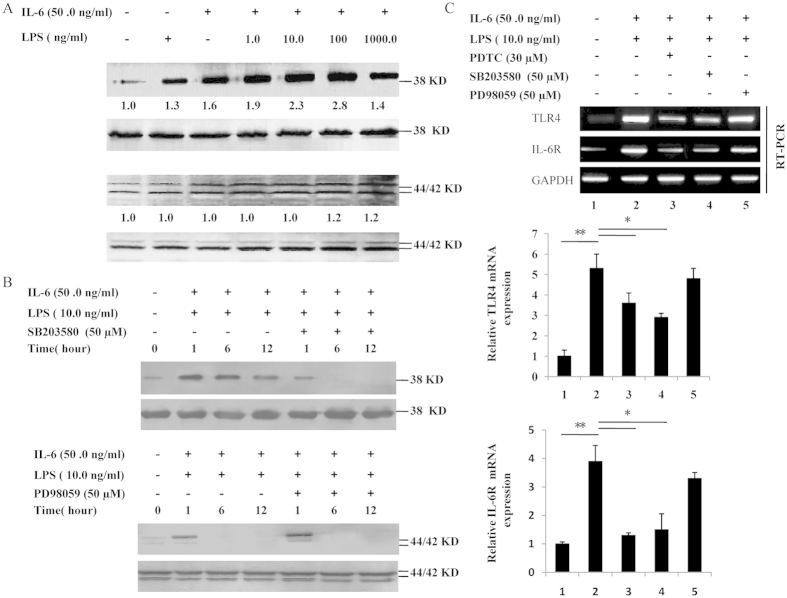
Phosphorylation of p38 MAPK and ERK1/2 and regulation of TLR4 and IL-6R mRNA expression. (**A**) Dami cells were treated with medium alone or varying concentrations of LPS (1.0-1000.0 ng/ml) and IL-6 (50.0 ng/ml) for 24 h. The levels of phosphorylation of p38 MAPK and ERK1/2 were determined by western blot using the specific antibodies P-p38 MAPK and P-ERK1/2, respectively. Equal loading in the lanes was evaluated by probing with the T-p38 MAPK and T-ERK1/2 antibodies. The value under each sample indicates the fold change of the protein level relative to that of the control. (**B**) The LPS-induced activation of ERK and p38 kinase in Dami cells was inhibited by specific inhibitors. Dami cells were pretreated with PD98059 or SB203580 at 50 μM for 30 min before stimulation with LPS and IL-6. The corresponding western blots for the total levels of this kinase are labeled T-ERK1/2 and T-p38 MAPK, and the control was used to ensure equal loading of the proteins. (**C**) LPS-induced TLR4 and IL-6R mRNA expression was inhibited by PD98059, SB203580 or PDTC. Dami cells were pretreated with or without PD98059, SB203580 or PDTC for 30 min and then stimulated with LPS plus IL-6 for 24 h. The relative TLR4 and IL-6R mRNA expression present in unstimulated cells is expressed as 100%. Similar results were observed in three independent experiments.

MAPKs/ERKs are key signal-transducing proteins that are involved in the inflammatory response and mediate cell proliferation and differentiation. In particular, the activation of ERK1/2 and p38 MAPK are involved in the TPO/Mpl pathways, as described in our previous study^25^,^26^. This finding prompted us to quantify the phosphorylation levels of ERK1/2 and p38 MAPK in the Dami cells following treatment IL-6 in combination with LPS at different time points. The results demonstrate that LPS induces the phosphorylation of p38 MAPK in a dose-dependent manner, with optimal induction using 10.0 to 100.0 ng/ml LPS in the presence of 50.0 ng/ml IL-6 (1.9- to 2.8-fold induction). However, the phosphorylation of ERK1/2 was not affected by LPS plus IL-6 treatment (Fig. 7A). Meanwhile, the kinetics of p38 MAPK phosphorylation was also analyzed in Dami cells. The phosphorylation of p38 MAPK was first detected 1 h after the addition of LPS, then peaked at 12 h and persisted up to 12 h. In contrast, the phosphorylation of ERK1/2 was detectable 1 h after IL-6 plus LPS stimulation and declined to almost basal levels by 6 h (Fig. 7B). We next sought to disrupt the ERK1/2 and p38 MAPK pathways via treatment with 50 μM PD98059 and 50 μM SB203580 before the LPS and IL-6 stimulations, respectively. As shown in Fig. 7C, SB203580 was more effective at inhibiting the LPS-induced activation of p38 MAPK than PD98059 was at inhibiting ERK1/2 activation in the presence of IL-6. These results collectively demonstrate that LPS induces the phosphorylation of p38 MAPK in a dose-dependent and time-dependent manner in Dami cell lines. To investigate the roles of the signaling pathways in TLR4 and IL-6R gene expression, Dami cells were pretreated with 50 mM PD98059, 50 mM SB203580, or 30 mM PDTC before LPS stimulation. As shown in Fig. 7C, only the SB203580 inhibitor and PDTC could suppress the LPS-induced up-regulation of TLR4 and IL-6R mRNA expression. These results indicate that p38 kinase is involved in the LPS-induced regulation of TLR4 and IL-6R gene expression in Dami cells.

### Effect of LPS and IL-6 on NF-κB activation and p65 DNA binding activities

NF-κB is a major regulatory component of the inflammatory responses mediated by LPS and pro-inflammatory cytokines. Earlier studies showed that the activation of IL-6 gene expression is associated with the NF-κB transcription factor^27^,^28^. We measured NF-κB transcriptional activity by using the pNF-κB-luc plasmid, which was generated by inserting four-spaced NF-κB binding sites into the pLuc-promoter vector. The Dami cells were transiently transfected with this plasmid and stimulated with 50.0 ng/ml IL-6, either with or without LPS. We found that pretreatment with IL-6 plus LPS 1.0 ng/ml condition produced the highest increase in IL-6-induced NF-κB-dependent luciferase activity compared with the IL-6 plus LPS 10.0 and 1000.0 ng/ml conditions (Fig. S1–A). An NF-κB p65 transcription factor assay was performed to further examine the effect of LPS and determine whether LPS alters the DNA binding activity of NF-κB. Figure S1–B shows that LPS (1.0 or 10.0 ng/ml) treatment for 48 h increased the binding activity of NF-κB p65 to the consensus DNA sequence. However, the 1000.0 ng/ml LPS treatment did not affect the DNA binding activity of NF-κB induced by IL-6 in a concentration-dependent manner. These results suggest that NF-κB activation plays an important role in the regulation of megakaryocytopoiesis.

### Effect of LPS with IL-6 on platelet production *in vivo*

**Figure 8:**
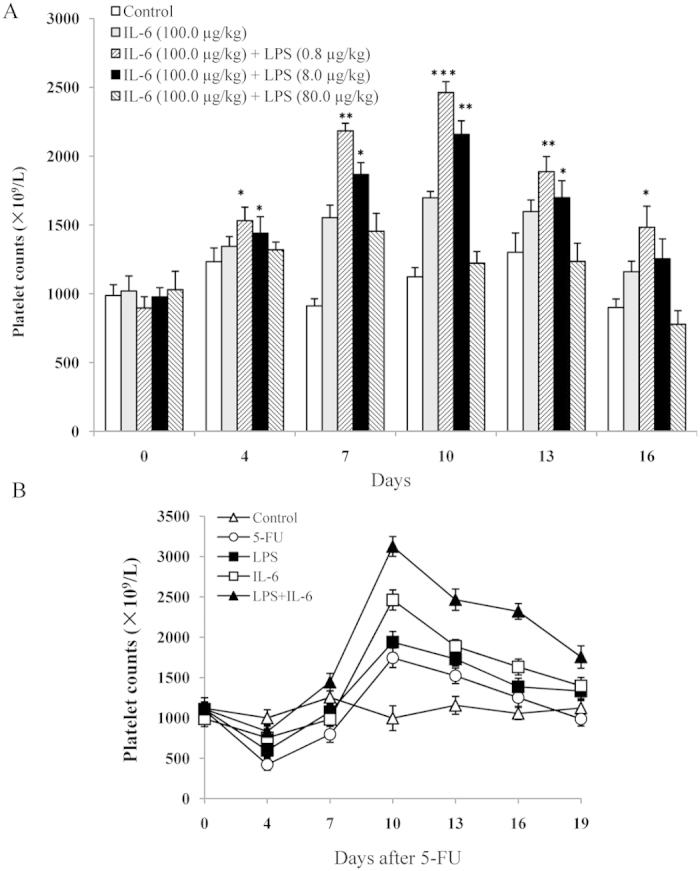
Effect of LPS or IL-6 alone or in combination on platelet counts. (**A**) Normal mice received IL-6 or IL-6 plus LPS at the indicated dosage once per day for 7 consecutive days. The control mice received PBS-BSA only. Platelets were counted at the indicated time points. Each dose resulted in significant increases in the platelet counts relative to the control (*P < 0.05; **P < 0.01, and ***P < 0.001). (**B**) Platelet counts in 5-FU-induced myelosuppressed mice. One hour after 5-FU injection at a dose of 150 mg/kg, the mice were intravenously given PBS-BSA (open circles), 8.0 μg/kg/day LPS (filled squares), 100 μg/kg/day IL-6 (open squares), or a combination of LPS and IL-6 (filled triangles). Mice untreated with 5-FU but still given PBS-BSA injections were used to gauge the baseline platelet levels (open triangles). Platelets were counted at the indicated time points.

To confirm the hypothesis that recombinant LPS with IL-6 can synergistically stimulate platelet production *in vivo*, we evaluated the platelet numbers in normal mice after LPS was administered. Figure 8A shows that LPS significantly increased the platelet counts between 0.8 and 8.0 μg/kg in a dose-dependent manner in the presence of 100.0 μg/kg IL-6. At 7 d, the platelet counts increased by 57.3%, 139.5%, and 104.7% with 100.0 μg/kg IL-6 alone and with 0.8 and 8.0 μg/kg LPS, respectively (*n* = 10, *P* < 0.01). At 10 d, the platelet counts increased by 51.2%, 119.3%, and 92.3% with 100.0 μg/kg IL-6 alone and with 0.8 and 8.0 μg/kg LPS, respectively (*n* = 10, *P* < 0.05). At each time point, the platelet counts observed in IL-6-treated mice were lower than in those treated with IL-6 and LPS (0.8 and 8.0 μg/kg), except in the presence of high LPS doses (80.0 μg/kg). The platelet numbers gradually declined at 16 d and returned to pre-injection levels. A second separate experiment was performed to evaluate the recovery of platelets after 5-FU-induced thrombocytopenia. As shown in Fig. 8B, with the exception of the control group, platelet nadirs were observed on days 4 and 7, and the depths of the nadirs were not significantly different among all groups after 5-FU injection. However, the recovery of platelet counts was faster after 7 d; the peak platelet levels occurred on day 10. The elevation of platelet levels in mice injected with LPS plus IL-6 was much higher than that in PBS-BSA-injected mice (*P* < 0.05). Mice treated with 8.0 μg/kg LPS plus 100.0 μg/kg IL-6 had a more marked response at day 10; the platelet levels in these mice were approximately 3123 ± 126 × 10^9^/L, compared with 1941 ± 131 × 10^9^/L for LPS treatment alone and 2463 ± 167 × 10^9^/L for IL-6 treatment alone (*n* = 10, *P* < 0.05). Recovery was not more pronounced in mice treated with IL-6 alone versus LPS alone (*P* > 0.05). After day 10, the platelet levels began to decline in all groups but did not return to the baseline values until 19 d. Overall, the platelet counts of two treatments and 5-FU group mice in different times were significantly different (ANOVA, *P* < 0.01, 0.05 and 0.01 for LPS plus IL-6, IL-6 and 5-FU respectively), while the control group in which time-of-day changes were not significant (ANOVA, *P* > 0.05). In addition, significant difference of platelet counts between different treatments was revealed by MNOVA (*P* < 0.01), showing as the platelet counts of mice treated by LPS plus IL-6 and IL-6 significantly higher than those of 5-FU treatment and control group.

## Discussion

LPS is the major physiologically active substance derived from bacteria, especially the Gram-negative bacteria frequently seen as part of the gut flora. During infection, the LPS released from bacteria can enter the host bloodstream and induce a strong immune response. The overuse of antibiotics could increase the LPS concentration in the bloodstream because lysed bacterial cells accumulate over a short time period^29^. However, traces of LPS have been detected in the human bloodstream; at a low concentration, LPS is required to maintain the normal immune functions of the human body^29^. In the present study, we propose the following model to illustrate how endogenous LPS links the gut microbiota to megakaryocytopoiesis in the presence of IL-6 (Fig. S2). The continuous release of the LPS in the intestine (e.g., through the killing of gut microbiota by kanamycin or through exogenous injection) increases gut permeability, which enhances the plasma LPS levels and activation of TLR4 signaling, which leads to the potentiated production of NF-κB and NF-κB p65 DNA binding activities. Meanwhile, up-regulated IL-6 could bind the increased IL-6R to augment p38 MAPK activation. Hyperactivation of p38 MAPK further up-regulates NF-κB activity, selectively promoting megakaryocyte development and platelet production (Fig. 8). Based on this regulation mechanism, the present therapies for enhancing the number of circulating platelets in cancer chemotherapy and thrombocytopenic patients should consider reasonable application of antibiotics so that the homestasis of gut microbiota could maintain endogenous LPS continuous release or adding the exdogenous LPS as complementary to the lost of its ability. Initially, we observed that the total number of platelets increased in the LPS plus kanamycin treatment group and with LPS alone. This increase in new platelets could be attributed to exogenous LPS because endogenous LPS release is eliminated by injecting kanamycin into the mice for 10 d, during which the original platelets, with a lifespan of approximately 7 d, could not survive (Fig. 1A). Moreover, we discovered that the number of circulating platelets decreased at 24 h post-LPS injection plus kanamycin (Fig. 1B). We hypothesized that LPS may contribute to platelet immune function by initially inducing platelet migration to the lung but later replenishing the blood platelet populations. This finding is consistent with those of a previous study, in which platelets contribute to the immune response in the pulmonary capillaries during sepsis in the blood^21^.

LPS is well known to induce the excess expression of cytokines, including IL-6, in the bone marrow cells, which functions as a potent promoter of megakaryocyte proliferation and differentiation^4^,^30^,^31^. We have previously reported that TPO regulation by the inflammatory mediator IL-6 could promote cell maturation during the later stages of megakaryocytopoiesis, which is consistent with results obtained by other investigators who suggest that although IL-6 has little capacity to induce megakaryocyte progenitor proliferation, it acts synergistically with the other cytokines and inducers to augment megakaryocyte colony formation^15^,^32^. These results suggest that LPS may promote megakaryocyte differentiation by inducing IL-6 and TPO expression in the entire hematopoietic system. Our results confirmed this hypothesis by evaluating both the mRNA expression and the serum levels of IL-6 and TPO in megakaryocytes during LPS stimulation (Fig. 2A,B). Therefore, we combined LPS and IL-6 to determine whether LPS and IL-6 synergistically induce megakaryocyte development and platelet production. The increased number of CD41-positive cells, along with the increased amount of CFU-MKs, indicated that the combined treatment with LPS and IL-6 greatly promoted the maturation and differentiation of megakaryocytes (Fig. 3) as well as the polyploidy of megakaryocytes (Fig. 4). Newly released platelets contain RNA and can be identified by thiazole orange staining, similar to erythroid cells^33^. We also found that the RP levels were higher at 100.0 μg/kg IL-6 plus 0.8 μg/kg LPS injection than at 100.0 μg/kg IL-6 plus 8.0 μg/kg or 80.0 μg/kg LPS injection (Fig. 5). The decrease in the thiazole orange signal following high-dose LPS treatment could be partially related to the inhibition of RNA synthesis in RPs. Thus, the positive effect of trace LPS plus IL-6 on the maintenance of RP production is optimal, suggesting that their synergistic effect could stimulate the formation of megakaryocytes and release of platelets. We determined the appropriate LPS doses via the dose-effect relationship of *in vitro* CFU-MK, and also tested the stimulating LPS concentration for increasing RPs and platelet production *in vitro*, which *in vitro*, as well as *in vitro*, it was observed that low concentrations LPS have greater effectiveness than high concentrations LPS. Furthermore, these LPS doses in mice suggest that the similar LPS produced in the human intestine, which is based on the mice body surface area was converted into the equal effective dose, may stimulate platelet production and maintain platelet numbers combined with the other cellular factors. To better understand the difference in LPS dose-effect relationship between mice and humans, we need to study the LPS concentration in human gut and serum and this justifies the need for the next experiment.

Multiple studies have shown that the presence of TLRs on megakaryocytes and platelets allows these cells to recognize bacterial proteins and regulate their immunity and function^27^,^34^,^35^. Although some studies most likely consider TLR4 on megakaryocytes to be a vestigial receptor, it could have a role in platelet production, suggesting that the same TLR4-expressing platelets are not limited to the innate immune system^36^,^37^. Moreover, platelets can interact with lung neutrophils and induce the formation of neutrophil extracellular traps, which catch bacteria in the capillary vessels^21^,^38^, where LPS released from bacteria could possibly have an effect on platelets. Although studies on the induced effects of LPS on megakaryocytes suggest that the effects are relatively less than expected, spleen cells from LPS-injected mice have been shown to produce more megakaryocyte colony-stimulating factor than the control mice^39^. Moreover, LPS increased the number of platelets and may increase their sensitivity to aggregation after 7 d, which corresponds to the platelet pool turnover period^17^. Considering that activating TLRs can influence cytokine secretion, we tested the effect of LPS on the production of its cytokine receptor using a real-time PCR assay. Upon stimulation, the patterns of IL-6R expression and the magnitude of expression varied depending on the stimulation of TLR4 by LPS in the presence of exogenous IL-6 (Fig. 6A). Notably, TLR4 could mediate a dose-dependent increase in IL-6R protein expression; in comparison, the stable TLR4 levels remained unchanged after IL-6R stimulation (Fig. 6B,C). In addition, IL-6 is possibly one of the downstream effector molecules that enables LPS to affect megakaryocyte maturation and production because IL-6 can interact with a variety of factors to regulate the growth of progenitor cells in late-stage hematopoiesis^40^. We also demonstrated that NF-κB activation and DNA-binding activities were remarkably altered over a wide range of LPS doses (Fig. S1); thus, NF-κB shares a similar LPS concentration-dependent pattern, as demonstrated by our CFU-MK stimulating studies (Fig. 3).

The mitogen-activated protein kinase (MAPK) family participates in transmitting extracellular signals to cytoplasmic and nuclear pathways^41^. In a subsequent study, we demonstrated that p38 kinase, rather than ERK1/2, was activated in Dami cells by LPS in dose- and time-dependent manner, suggesting that the phosphorylation of ERK and p38 is regulated by different mechanisms and that p38 kinase plays the key role in the response of Dami cells to LPS stimulation (Fig. 7A,B). Conversely, SB203580 and PDTC prevented the increase in TLR4 and IL-6R mRNA (Fig. 7C), suggesting that both the MAPK and NF-kB signaling pathways participate in the regulation of TLR and IL-6R gene expression. Thus, the up-regulation of TLR4 and IL-6R expression by LPS might promote the overall responses of megakaryocytes to inflammatory factors and help explain the synergy between LPS and IL-6 in the induction of platelet production signaling. The presence of LPS plus IL-6 in the sera provided another explanation for the observation of the residual level of platelets in TPO^-/-^ mice, which were not more vulnerable to hemorrhage than normal mice and retained approximately 15% of the circulating platelets and 15% of the marrow and spleen megakaryocytes^42^,^43^. Under normal conditions LPS and IL-6 may synergistically interact to stimulate megakaryocyte maturation given that normal *in vivo* TPO levels are very low (50–150 pg/ml)^20^. Thus, IL-6 may have secondary effects on the terminal stages of megakaryocyte maturation, which require the appropriate dose of LPS for triggering many of the same responses as TPO. Although IL-6 is considered to be one of the active factors regulating platelet formation, our *in vivo* data indicate that the administration of lower-dose LPS plus IL-6 to normal mice increased the number of blood platelets to a much higher degree compared with IL-6-treated mice (Fig. 8A). However, high-dose LPS reduced platelet counts in normal mice (Fig. 8A), which suggests that the reverse effect was due to excessive platelet aggregation in the whole blood via the activation of the TLR4/IL-6R pathway and the modulation of platelet reactivity. In an attempt to mimic chemotherapy treatments, the mice were subjected to 5-FU injections, which resulted in a drastic reduction in platelet numbers. Treatment with 8.0 μg/kg LPS alone or 100.0 μg/kg IL-6 alone did not enhance the platelet recovery compared with the control after 5-FU injection. A combination of LPS and IL-6 resulted in higher levels of platelet recovery after the 5-FU-induced thrombocytopenia (Fig. 8B), indicating that their additive effects are greater than those observed in platelet recovery in thrombocytopenic mice compared with normal mice. One possible explanation might be that IL-6 may only minimally raise platelet numbers but that at the critical moment when platelet numbers drop below safe threshold levels, LPS would effectively compensate for the decrease in platelets induced by 5-FU.

In summary, our data demonstrate that the observed additive effects of LPS and IL-6 not only greatly enhanced murine megakaryocyte proliferation but also enhanced megakaryocyte maturation *in vitro* and platelet formation *in vitro.* Moreover, we provide new insights into the cross-regulation of TLR4/IL-6R signaling, along with the specific up-regulation of IL-6 production and activation of NF-κB and p38 MAPK mediated by a trace amount of LPS plus IL-6. Our findings raise the possibility of the coordinated use of LPS and IL-6 as a possible future therapeutic strategy in a variety of thrombocytopenic events, in which mice are sensitive to LPS as a broader simulator, and regulating IL-6 to induce the megakaryocyte response and platelet production.

## Additional Information

**How to cite this article**: Wu, D. *et al.* Micro-concentration Lipopolysaccharide as a Novel Stimulator of Megakaryocytopoiesis that Synergizes with IL-6 for Platelet Production. *Sci. Rep.* **5**, 13748; doi: 10.1038/srep13748 (2015).

## Supplementary Material

Supplementary Information
