# Supplementary material for: Micro-concentration Lipopolysaccharide as a Novel Stimulator of Megakaryocytopoiesis that Synergizes with IL-6 for Platelet Production
Source: Sci Rep. 2015 Sep 2;5:13748. doi: 10.1038/srep13748 (PMC4557119; doi:10.1038/srep13748)
Supplement: Supplementary Information [file srep13748-s1.pdf]

Supplementary information

Micro-concentration Lipopolysaccharide as a Novel Stimulator of Megakaryocytopoiesis that Synergizes with IL-6 for Platelet Production

Di Wu, Jun Xie, Xuejun Wang, Bingcheng Zuo, Yin Yu, Tao Jing, Songmei Zhang, Qing Zhang

Figure S1 | Effects of LPS on IL-6-induced NF-κB transcriptional activity and NF-κB-DNA binding activity. (A) Cells were transiently transfected with a pNF-κB-luc reporter construct with the phRL-TK vector as an internal control. After transfection, the cells were pretreated with IL-6 either alone or in combination with LPS (0.1, 10, and 1000 ng/ml) for 48 h, and luciferase activity was measured. (B) The effect of LPS plus IL-6 on the NF-κB p65 binding activity in the nuclear extracts from 293T cells was measured using the ELISA-based NF-κB assay, as described in the Methods section. The experiments were repeated three times, and similar results were obtained.

Figure S2 | Proposed model for the regulation of thrombocytopoiesis via the LPS/IL-6 signaling pathway. Activation of TLR4 by the administration of LPS or release of LPS from the gut microbiota potentiates the production of NF-κB and enhances the NF-κB p65 DNA binding activity. Meanwhile, the up-regulated IL-6 can bind the increased IL-6R to initiate an autocrine loop and mediate p38 MAPK activation. The up-regulation of these costimulatory molecules effectively activates megakaryocytopoiesis and platelet production through distinct but overlapping TLR4- and IL-6R-dependent molecular mechanisms.

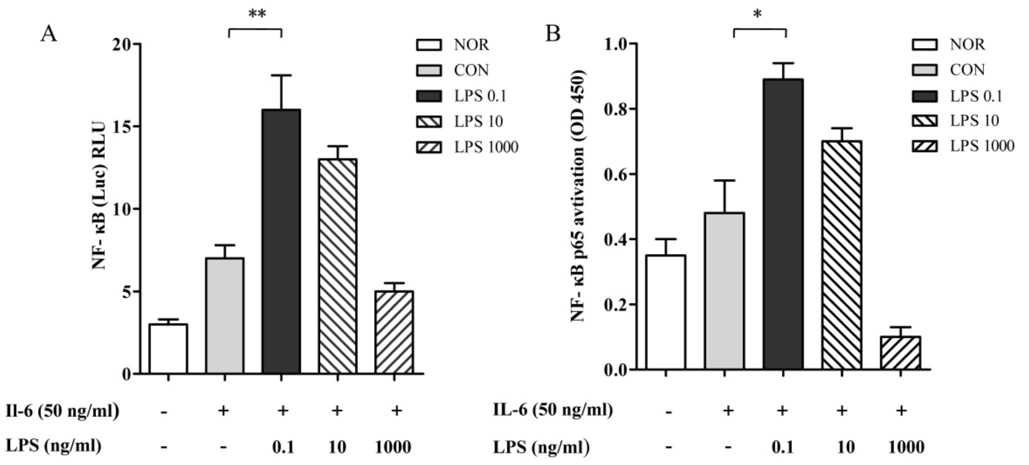
A
**
NF- κB (Luc) RLU
20
15
10
5
0
NOR
CON
LPS 0.1
LPS 10
LPS 1000
Il-6 (50 ng/ml)
LPS (ng/ml)
0.1
10
1000
B
*
NF- κB p65 avtivation (OD 450)
1.0
0.8
0.6
0.4
0.2
0.0
IL-6 (50 ng/ml)

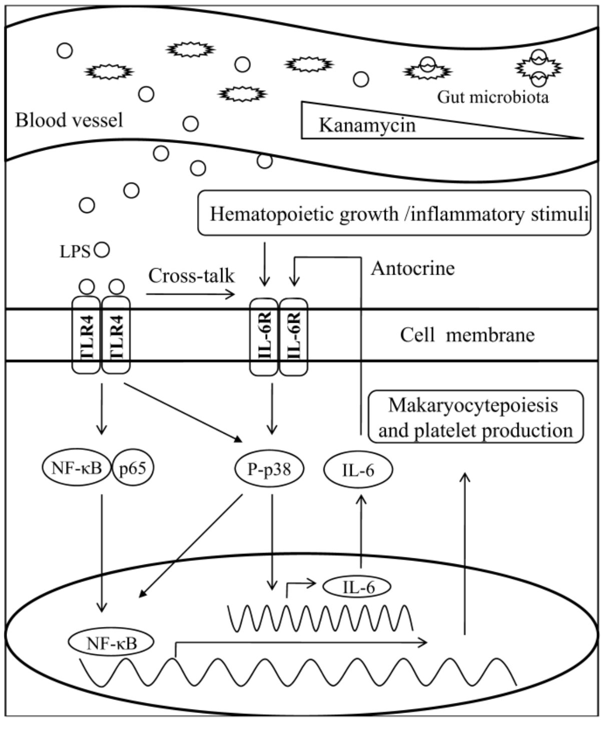
Blood vessel
Gut microbiota
Kanamycin
Hematopoietic growth /inflammatory stimuli
LPS
Antocrine
Cross-talk
Cell membrane
TLR4
IL-6R
Makaryocytepoiesis
and platelet production
NF-κB
p65
P-p38
IL-6
